# Knowledge-based recommender systems: overview and research directions

**DOI:** 10.3389/fdata.2024.1304439

**Published:** 2024-02-26

**Authors:** Mathias Uta, Alexander Felfernig, Viet-Man Le, Thi Ngoc Trang Tran, Damian Garber, Sebastian Lubos, Tamim Burgstaller

**Affiliations:** ^1^Siemens Energy AG, Erlangen, Germany; ^2^Institute of Software Technology (IST) - Applied Software Engineering & Ai Research Group (ASE), Graz University of Technology, Graz, Austria

**Keywords:** recommender systems, semantic recommender systems, knowledge-based recommender systems, case-based recommendation, constraint-based recommendation, critiquing-based recommendation, constraint solving, model-based diagnosis

## Abstract

Recommender systems are decision support systems that help users to identify items of relevance from a potentially large set of alternatives. In contrast to the mainstream recommendation approaches of collaborative filtering and content-based filtering, knowledge-based recommenders exploit semantic user preference knowledge, item knowledge, and recommendation knowledge, to identify user-relevant items which is of specific relevance when dealing with complex and high-involvement items. Such recommenders are primarily applied in scenarios where users specify (and revise) their preferences, and related recommendations are determined on the basis of constraints or attribute-level similarity metrics. In this article, we provide an overview of the existing state-of-the-art in knowledge-based recommender systems. Different related recommendation techniques are explained on the basis of a working example from the domain of survey software services. On the basis of our analysis, we outline different directions for future research.

## 1 Introduction

Recommender systems support users in identifying relevant items from a large set of alternatives and thus help to reduce the complexity of decisions and increase user satisfaction and sales (Felfernig and Burke, 2008; Burke et al., 2011; Knijnenburg et al., 2012). *Single-shot recommender systems* recommend items based on already stored preferences of a user (e.g., videos a user purchased/liked in the past) without the need of engaging the user in a dialog for capturing further preferences (Rafter and Smyth, 2005; Pramod and Bafna, 2022). In contrast, *conversational recommender systems* perform preference elicitation on the basis of a user/recommender dialog (Goeker and Thompson, 2000; Bridge, 2002; Gao et al., 2021). Depending on the application scenario, different recommendation approaches can be applied (see Tables 1, 2 for an overview of used data sources and properties of those recommendation approaches).

**Table 1:** Basic recommendation approaches of collaborative filtering (CF), content-based filtering (CBF), and knowledge-based recommendation (KBR), and used data sources including the current user preferences, a user's preference history, the preferences of nearest neighbors, and item knowledge.

**Approach**	**Preferences**	**Preference history**	**Nearest neighbors**	**Item knowledge**
Collaborative (CF)	–	×	×	–
Content-based (CBF)	–	×	–	×
Knowledge-based (KBR)	×	–	–	×

**Table 2:** *Basic properties* of different recommendation approaches: *easy setup* = low effort needed for setting up the recommender system, *dialog-based* = conversational process between system and user, *serendipity* = effect of proposing unexpected but relevant recommendations, *cold-start problem* = initial data are needed to provide reasonable recommendations, *high-involvement items* = a user carefully evaluates the candidate items since suboptimal decisions can have significant negative consequences.

**Recommendation approach**	**Collaborative (CF)**	**Content-based (CBF)**	**Knowledge-based (KBR)**
Easy setup	×	×	–
Dialog-based	–	–	×
Serendipity	×	–	×
Cold-start problem	×	×	–
High-involvement items	–	–	×

*First*, collaborative filtering (CF) (Ekstrand et al., 2011) is based on the idea of simulating word-of-mouth promotion by determining recommendations on the basis of the preferences of so-called nearest neighbors (NNs), which are users with preferences similar to those of the current user. For example, a movie that has been watched by the nearest neighbors of the current user (but not by the current user) and has been evaluated positively by those nearest neighbors should also be recommended to the current user. A major advantage of CF approaches is an easy setup since no detailed information about the provided items is needed. Furthermore, serendipity effects can be created, i.e., collaborative filtering is a good choice for finding recommendations which are (in a positive sense) a kind of “surprise” for the user, thus supporting diversity and the identification of completely unexpected/unsearched items (Iaquinta et al., 2008; Ziarani and Ravanmehr, 2021). CF typically follows the idea of single-shot recommendation where no session-specific user dialogs are needed. A disadvantage of CF recommenders is cold-start problems which are due to the sparsity of rating information (regarding users and items) available in the recommendation algorithm, making it hard to identify accurate recommendations (Lika et al., 2014).

*Second*, content-based filtering (CBF) (Pazzani and Billsus, 2007) is based on the idea of recommending items which are in one way or another similar to items the user has consumed in the past. In contrast to CF, CBF does not exploit the preferences of nearest neighbors which limits the cold-start problem to the availability of initial user item ratings. A kind of disadvantage of CBF recommendation approaches is need of item knowledge, for example, in terms of item categories and/or item descriptions which are used to derive a user profile representing a user item preference history. CBF systems are typically of type “single-shot”, i.e., no conversational user interface needs to be provided for preference elicitation purposes. *Basic CBF* that purely focuses on the recommendation of similar items is less appropriate when it comes to the triggering of serendipity effects (Iaquinta et al., 2008). For example, if a user has purchased the song *Personal Jesus* from *Depeche Mode*, an item that could be recommended in the future could be the same song performed by *Johnny Cash*. However, further developments in content-based recommendation have improved the capability of CBF with regard to the achievement of serendipity effects, for example, on the basis of increasing the diversity of recommended items (Maccatrozzo et al., 2017). In the line with CF approaches, CBF has an easy setup process, since only item knowledge is needed in the setup phase. Since preferences change over time, both CF and CBF have an issue with item domains characterized by infrequently articulated and changing preferences. For example, time intervals between individual car purchases of a user could be around 10 years—in such long time periods, user preferences could completely change, which makes the identification of recommendations challenging without using a preference elicitation dialog.

*Third*, knowledge-based recommender (KBR) systems can be considered as complementary to CF- and CBF-based approaches in terms of avoiding the related cold-start difficulties (Burke, 2000; Towle and Quinn, 2000; Lorenzi and Ricci, 2005). KBR systems are based on the idea of collecting the preferences of a user (preference elicitation) within the scope of a dialog and then to recommend items either (1) on the basis of a predefined set of recommendation rules (constraints) or (2) using similarity metrics that help to identify items which are similar to the preferences of the user. The first approach is denoted as *constraint-based recommendation* (Felfernig and Burke, 2008), whereas the second one is referred to as *case-based recommendation* (Lorenzi and Ricci, 2005)—these two can be regarded as major types of knowledge-based recommender systems (Aggarwal, 2016). Serendipity effects in knowledge-based recommendation are limited by the static encoding in terms of constraints (rules) and similarity metrics. KBR systems support the determination of recommendations specifically in complex and high-involvement item domains [domains where suboptimal decisions can have significant negative consequences, for example, when investing in high-risk financial services (Felfernig et al., 2006)] where items are not bought on a regular basis (Aggarwal, 2016). Example item domains are financial services (Felfernig et al., 2007; Musto et al., 2015), software services (Felfernig et al., 2021), apartment or house purchasing (Fano and Kurth, 2003), and digital cameras (Felfernig et al., 2006). These systems are able to take into account constraints (e.g., high-risk financial services must not be recommended to users with a low preparedness to take risks) and provide explanations of recommendations also in situations where no solution could be identified. In contrast to CF and CBF, KBR systems support explicit preference elicitation dialogs which makes them immune with regard to user preferences changing over time. Due to an often time-intensive knowledge exchange between domain experts and knowledge engineers, the definition of recommendation knowledge can trigger high setup costs (Ulz et al., 2017). Both types of KBR systems (case-based and constraint-based) are conversational, since a preference elicitation dialog helps to figure out user preferences (Gao et al., 2021).

### 1.1 Further recommendation approaches

In addition to the three basic approaches of collaborative filtering, content-based filtering, and knowledge-based recommendation, there exist various further approaches. First, *hybrid recommender systems* (Burke, 2002) exploit synergy effects by combining different recommendation approaches. For example, by combining CBF with CF, the cold-start problem can be solved by initially applying CBF in order to collect the relevant rating data needed for establishing a CF-based approach. Furthermore, *group recommender systems* (Masthoff, 2015; Dara et al., 2020; Felfernig et al., 2024b) support the determination of recommendations for whole groups, i.e., not individual users. These systems apply basic recommendation approaches in such a way that recommendations satisfy the preferences of all or at least a subset of the group members. For example, the *average* user rating per item can be regarded as an item rating provided by the whole group.

### 1.2 Article contributions

The major contributions of this article are as follows. First, we provide an overview of the existing state-of-the-art in knowledge-based recommender systems. Our work significantly enhances existing overviews on the same topic (see the last row of Table 3) specifically in terms of the inclusion of new technological approaches and a broader view on how knowledge-based technologies are applied to recommender systems. Second, to assure understandability, we explain different underlying recommendation techniques on the basis of a working example. Third, to foster further related work, we discuss different open research directions derived from our literature analysis.

**Table 3:** Overview of scientific contributions in knowledge-based recommender systems organized in the line of the primary focus of the paper: ALG, algorithms; PREF, preference elicitation; KA, knowledge acquisition; APP, application; FUR, further recommendation approaches and knowledge representations; OV, overview articles/papers including aspects related to knowledge-based recommendation.

**Approach**	**ALG**	**PREF**	**KA**	**APP**
Case-based (CBR)	Jannach, 2006	Goeker and Thompson, 2000; Bridge, 2002; McSherry, 2003; Mirzadeh et al., 2005; Christakopoulou et al., 2016	Khan and Hoffmann, 2003; Zou et al., 2020	Fesenmaier et al., 2003; Lee and Kim, 2015; Musto et al., 2015; Feely et al., 2020; Bokolo, 2021; Hernandez-Nieves et al., 2021
Critiquing-based (CRIT)	Reilly et al., 2004; Smyth et al., 2004; McCarthy et al., 2005; Mandl and Felfernig, 2012; Murti et al., 2016	Zhang et al., 2008; Chen et al., 2017; Xie et al., 2018; Wu et al., 2019; Güell et al., 2020	McCarthy et al., 2010	Burke et al., 1996; Grasch et al., 2013
Constraint-based (CON)	Cöster et al., 2002; Felfernig and Burke, 2008; Falkner et al., 2011; Fargier et al., 2016; Erdeniz et al., 2019; Teppan and Zanker, 2020; Felfernig et al., 2023a	Towle and Quinn, 2000; Fano and Kurth, 2003; Junker, 2004; Felfernig et al., 2009b, 2012, 2013c, 2018a,b; Pereira et al., 2016; Tazl et al., 2019; Erdeniz et al., 2022; Uta et al., 2022	Jannach and Kreutler, 2007; Felfernig et al., 2009a, 2013a,b, 2015; Daoudi et al., 2016; Uta et al., 2021; Lubos et al., 2023	Felfernig and Kiener, 2005; Felfernig et al., 2006, 2007; Zanker et al., 2006; Murphy et al., 2015; Wobcke et al., 2015; Ulz et al., 2017; Almalis et al., 2018
Further (FUR)	McCarthy et al., 2006	Burke, 2002; Zhou et al., 2020; Zhu et al., 2020; Le et al., 2022	Wang et al., 2019a,b; Sun et al., 2020; Esheiba et al., 2021; Sha et al., 2021	Lee et al., 2006; Bahramian and Ali Abbaspour, 2015; Colombo-Mendoza et al., 2015; Pessemier et al., 2017; Cordero et al., 2020; Dong et al., 2020
Overviews (OV)	Bridge et al., 2005; Felfernig et al., 2011, 2024b; Aggarwal, 2016; Gao et al., 2021	Lorenzi and Ricci, 2005; Chen and Pu, 2012; Masthoff, 2015; Atas et al., 2021; Felfernig et al., 2021; Jannach et al., 2021; Tran et al., 2023	Felfernig, 2007; Bouraga et al., 2014; Felfernig et al., 2014; Cena et al., 2021; Popescu et al., 2022	Burke, 2000; Felfernig et al., 2024a

The remainder of this article is organized as follows. In Section 2, we explain the methodology applied for our literature analysis. An introduction to basic knowledge-based recommendation approaches is presented in Section 3, where we specifically focus on techniques and applications of case-based and constraint-based recommendation. Thereafter, in Section 4, we provide an overview of advanced techniques in knowledge-based recommendation with a specific focus on integration scenarios with machine learning. In this context, we also discuss related topics such as hybrid recommendation with knowledge-based recommenders and knowledge-based group recommender systems. In Section 5, we discuss the identified research directions. The article is concluded with Section 6.

## 2 Methodology

Our overview of the existing state-of-the-art in knowledge-based recommendation (KBR) is based on a literature analysis conducted using the activities of *search* (querying of leading research portals), *review* (evaluating and classifying the identified scientific contributions), and *discussion of reviewed contributions* (deciding about inclusion of the identified studies in our overview based on the selection criteria of quality and relevance). Queries have been performed between December 2023 and January 2024 on the research platforms Google Scholar,^1^ ResearchGate,^2^ ScienceDirect,^3^ SpringerLink,^4^ and Elsevier,^5^ using the keywords of “recommender systems” + [“knowledge-based” | “case-based” | “critiquing” | “ontologies” | “knowledge graphs” | “constraint satisfaction” | “conversational” | “mass customization” | “groups” | “overview”], representing 10 individual search queries. Using these queries, we have also analyzed topic-related scientific contribution in conferences and journals: the International Joint Conference on Artificial Intelligence (IJCAI), the AAAI Conference on Artificial Intelligence, the European Conference on Artificial Intelligence (ECAI), the Recommender Systems Conference (RecSys), the User Modeling, Adaptation, and Personalization (UMAP) conference, the Constraint Programming Conference (CP), the Software Product Line Conference (SPLC), User Modeling and User-Adapted Interaction (UMUAI), and ACM Transactions on Recommender Systems. Thereafter, using the snowballing technique (Wohlin, 2014), we have analyzed the reference sections of the identified studies. Major criteria for estimating the relevance of a paper were (1) a *topic-wise match*, i.e., a relationship to knowledge-based recommenders and (2) an *official publication* in a workshop, conference, journal, magazine, book, or PhD thesis. With our analysis, we have identified 97 publications directly related to the topic of knowledge-based recommendation that have been used as a basis for this article (see Table 3).

## 3 Basic approaches and applications

In this section, we introduce a working example from the domain of *survey software service recommendation* that is used to explain different knowledge-based recommendation approaches. The underlying scenario is that users search for a survey software configuration that fulfills their preferences, for example, a researcher received a new project funding and is now interested in purchasing a survey software that supports different user studies envisioned for the new project. In many cases, Web applications (services) are preferred over solutions requiring an installation at the customer site. In such a scenario, knowledge-based recommenders can support users in identifying an appropriate configuration (including pricing) of a survey software service that fulfills their wishes and needs. In our example, we include the user-selectable survey software features *ABtesting* (should ABtesting be supported by the survey software), *statistics* (should a basic statistics feature be included), *multiplechoice* (should the specification of multiple-choice questions be possible), and *license* [which license model is preferred—*free of charge* (0) vs. *with costs*, i.e., 100]. In the following, we show how knowledge-based recommenders can support the identification of relevant items, i.e., survey software configurations.

Knowledge-based recommender systems can operate on different knowledge representations—these knowledge representations will be explained and exemplified in Section 3.1. Thereafter, we introduce the two basic approaches to knowledge-based recommendation, which are (1) *case-based recommendation* including *critiquing-based recommendation* as a specific form of case-based recommendation (see Section 3.2) and (2) *constraint-based recommendation* (see Section 3.3).

### 3.1 Recommendation knowledge representations

Knowledge representations of knowledge-based recommender systems can be (1) *table-based* which is used in scenarios where items are represented in terms of product table entries or (2) *constraint-based* which is used in scenarios where items are defined on the basis of a set of *restrictions* (also denoted as *rules* or *constraints*). In the first case (extensional representation—see, for example, Table 4), each item that could be recommended is explicitly defined in a corresponding item (product) table. In the second case, there is no need to enumerate all items since items are specified in a constraint-based fashion (intensional representation—see, for example, Table 5).

**Table 4:** Extensional itemset representation where each item is represented as a table entry assuming the item (table) attributes *ABtesting* (0,1), *statistics* (0,1), *multiplechoice* (0,1), and *license* (0,100).

**Item**	**ABtesting**	**Statistics**	**Multiplechoice**	**License**
*i*_1_	0	1	0	0
*i*_2_	0	0	1	100
*i*_3_	1	1	0	100
*i*_4_	0	1	1	100
*i*_5_	1	1	1	100

In our working example, the offered survey software configurations (items *i*_1_..*i*_5_) represent the complete set of items (i.e., the product/item catalog). For example, item *i*_1_ represents a survey software configuration (a “free of charge version”) which does not support *ABtesting* and *multiplechoice* questions, i.e., only single choice questions can be asked and the *statistics* feature supports a basic analysis of survey data.

**Table 5:** Intensional solution space representation in terms of a set of constraints {*c*_1_..*c*_5_} on the CSP variables *ABtesting, statistics, license*, and *multiplechoice*.

**Id**	**Constraint**
*c*_1_	ABtesting=1 → statistics=1
*c*_2_	¬(ABtesting=1 ∧ license=0)
*c*_3_	¬(multiplechoice=1 ∧ license=0)
*c*_4_	¬ multiplechoice=1 ∧¬ ABtesting=1 → license=0
*c*_5_	¬(ABtesting=0 ∧ statistics=0 ∧ multiplechoice=0 ∧ license=0)

#### 3.1.1 Table-based representations

In many knowledge-based recommendation scenarios, the offered itemset is represented in terms of an item (product) table (see Table 4). The itemset is defined extensionally, i.e., all selectable alternatives (items) are enumerated. In our example, five different service configurations (items *i*_1_..*i*_5_) can be offered to a user. Table-based representations can be applied if the set of offered items is limited, i.e., the item space is rather small which is often the case, for example, in digital camera or financial service recommendation (Felfernig et al., 2006, 2007). Using a table-based knowledge representation, corresponding database queries can be performed to identify a set of recommendation candidates that support the preferences defined by the user (Felfernig et al., 2006, 2023a). An example of a *user preference* regarding the itemset defined in Table 4 could be *ABtesting* = 1, meaning that the user is interested in a survey software that includes (supports) *ABtesting*. For simplicity, we assume that attributes have a corresponding Boolean domain definition, for example, the domain of attribute *ABtesting* is {0, 1} where 1 = *true* (feature included) and 0 = *false*. One exception thereof is the *license* attribute with a domain {0, 100} representing the price of a license.

#### 3.1.2 Constraint-based representations

Alternatively, itemsets can be represented in an intensional fashion on the basis of a set of domain-specific constraints (see Table 5), for example, as a *constraint satisfaction problem* (CSP) (Rossi et al., 2006; Felfernig and Burke, 2008). Such knowledge representations are specifically useful if the solution (item) space becomes intractable, i.e., defining and maintaining all alternatives is extremely inefficient and error-prone (or even impossible) and related search queries become inefficient and at least impractical for interactive settings (Falkner et al., 2011).

The constraints in Table 5 represent exactly the solution space, as shown in Table 4. If such constraints are not explicitly known, a given set of items can be defined in terms of one constraint in disjunctive normal form, for example, the entries in Table 4 would be represented as (*ABtesting* = 0∧*statistics* = 1 ∧ *license* = 0 ∧ *multiplechoice* = 0) ∨ .. ∨ (*ABtesting* = 1 ∧ *statistics* = 1 ∧ *license* = 100 ∧ *multiplechoice* = 1)—for details, we refer to Felfernig and Burke (2008).

#### 3.1.3 Further knowledge representations

Intensional representations of solution spaces (itemsets) as presented in Table 5 can be implemented with different knowledge representations ranging from *constraint satisfaction problems* (CSPs) (Rossi et al., 2006; Felfernig and Burke, 2008) and *Boolean satisfiability problems* (SAT problems) (Biere et al., 2021; Felfernig et al., 2021) to less frequently used recommendation knowledge representations such as *answer set programming* (ASP) (Eiter et al., 2009; Teppan and Zanker, 2020) and *ontology-based knowledge representations*, for example, *description logics* (DL) (Lee et al., 2006; McGuinness, 2007). In addition, database queries can be applied in such a way that intensionally formulated recommendation knowledge is encoded directly in database queries—see, for example, Felfernig et al. (2006, 2023a). Without loss of generality, in this article, we focus on CSP-based representations when discussing the concepts of constraint-based recommendation. As a basis for our discussion of case-based recommendation approaches, we will use the entries of Table 4.

### 3.2 Case-based recommendation

#### 3.2.1 Basic approach

Following the basic concepts of case-based reasoning (Kolodner, 2014), case-based recommendation uses a knowledge-rich representation of the item domain and can therefore be classified as a kind of knowledge-based recommendation (Burke, 2000; Khan and Hoffmann, 2003; Lorenzi and Ricci, 2005). In a basic setting, the item assortment can be regarded as the case base, and recommendations are determined by identifying those items from the case base which “support” the user preferences (Lorenzi and Ricci, 2005). In this context, product features (also denoted as properties or attributes) are used to specify user preferences. The more preferences are supported by an item, the higher its user relevance. Examples of case-based recommendation approaches going beyond an equality match between item properties and user preferences are the following—see, for example, Lorenzi and Ricci (2005).

#### 3.2.2 Interest confidence value

This approach is based on the idea of predicting item relevance on the basis of the similarity of a new item with items a user has already evaluated positively in the past (Lorenzi and Ricci, 2005; Musto et al., 2015). This approach is in the line with the ideas of content-based recommendation where the similarity between a new item and properties of already consumed items is used to estimate recommendation relevance. A simple example of the application of interest confidence values (*icv*) is shown in Table 6, where the *average* (*avg*) *similarity* between a new item (the user did not see up to now) and (consumed) items positively evaluated in the past is used to estimate item relevance.

**Table 6:** Example of an *interest confidence value* (*icv*)-based evaluation of the user relevance of new items (*in*_*k*_) on the basis of the similarity with already consumed items *i*_*l*_.

**Consumed items**	**New items**				
	*in*_1_	*in*_2_	*in*_3_	*in*_4_	*in*_5_
*i*_1_	0.2	0.4	0.3	0.9	0.5
*i*_2_	0.4	0.6	0.2	0.6	0.7
*i*_3_	0.4	0.1	0.5	0.5	0.6
icv (avg)	0.33	0.36	0.33	0.66	0.6

For example, the similarity between the consumed item *i*_1_ and the new item *in*_2_ is assumed to be 0.4. In this example, item *in*_4_ has the highest *interest confidence value* (0.66) and therefore can be regarded as a user-relevant item.

#### 3.2.3 Attribute-level similarity metrics

This approach is based on the idea that the degree of satisfaction of individual user requirements has a direct impact on the corresponding item ranking (Lorenzi and Ricci, 2005). In such contexts, attribute-level similarity metrics (McSherry, 2003) can be used to determine the similarity between the user requirements and the items included in the product assortment. For example, if explicit price information of an item is available, the *less is better* (LIB) similarity metric can be applied (the lower the price, the higher the item ranking). The *more is better* (MIB) similarity metric can be applied in the context of technical attributes, such as the resolution of a digital camera or the return rate of a financial service (McSherry, 2003; Felfernig et al., 2013c) (the higher the attribute value the better). *Equal is better* (EIB) is used in contexts where users explicitly require a specific attribute value (e.g., the color of a car), and *nearer is better* (NIB) can be used in situations where an item should fulfill a specific requirement as good as possible, for example, the size of a TV screen in the living room. The overall similarity between user requirements and an item is then determined on the basis of a “global” similarity that combines individual attribute-level similarity functions. In our working example, the *license* attribute could be associated with an *LIB*, the remaining attributes with an *EIB* attribute-level similarity.

#### 3.2.4 Refine, relax, and compromise

When querying a case base (i.e., an item catalog) with a set of user preferences, it can happen that the number of candidate items (items that satisfy the user preferences) is (1) too large and—as a consequence—the initial set of user requirements needs to be refined or (2) too small (in the worst case, no solution could be identified), which means that the user requirements have to be relaxed. In the first case, the set of user requirements needs to be extended, for example, if a user has specified the requirement *statistics* = 1, this results in four candidate items ({*i*_1_, *i*_3_, *i*_4_, *i*_5_}—see Table 4). Refining the original query to *statistics* = 1 ∧ *ABtesting* = 1 reduces the set of candidate items to {*i*_3_, *i*_5_}. In contrary, if a user has specified the requirement *ABtesting* = 1 ∧ *license* = 0, the corresponding query would result in an empty set. The user has two options to resolve the inconsistency, i.e., to relax the query: either to exclude *ABtesting* or to accept a *license* payment, i.e., *license* = 100. Such trade-off decisions can be supported by so-called *compromise-based user interfaces* which group items with regard to different possible compromises (McSherry, 2003). Such interfaces would group and explain candidate items in the line of, for example, *most of your requirements are fulfilled, however, these items require a license payment*.

#### 3.2.5 Critiquing

Critiquing-based recommendation originates from different case-based recommenders (Kolodner, 1992; Bridge et al., 2005) which often support a search-based approach where—depending on a set of defined user preferences—the system recommends items which support in one way or another those preferences. While basic case-based and constraint-based recommendation focus on supporting a *search-based recommendation process*, critiquing-based recommender systems (as a specific type of case-based recommender system) follow a *navigation-based approach* (Chen and Pu, 2012), which focuses on better supporting users in *exploring* the solution (item) space. Critiquing-based recommendation is based on the idea of presenting *example (reference) items* to a user who then can (1) accept the proposed item or (2) define critiques in terms of changes that are needed to make an item acceptable. Existing critiquing-based recommenders are based on a predefined itemset (product table)—see Table 4. These systems are regarded as knowledge-based, since items are associated with semantic properties, and user-defined (selected) critiques can be regarded as logical criteria (constraints) to be fulfilled by recommendations.

#### 3.2.6 Unit critiquing

Let us assume that in the context of our working example a critiquing-based recommender supports six basic critiques which are (1) *furtherstat*, (2) *reducestat*, (3) *excludemc*, (4) *includemc*, (5) *excludelicense*, and (6) *includelicense*. The critiquing-based approach used in this example is *unit-critiquing* (Chen and Pu, 2012; Mandl and Felfernig, 2012), where in each critiquing cycle, a critique on an individual item attribute is specified. Table 7 shows how critiques can be translated into corresponding item selection criteria. For example, if a user selects the critique *further statistics* (*furtherstat*) and statistics is not included in the shown reference item, the corresponding selection criteria would be *statistics* = 1. Furthermore, if statistics is already included in the reference item, *ABtesting* = 1 would be defined as additional selection criteria (see the entries in Table 7).

**Table 7:** Translating critiques into item selection criteria where *mc*=*multiplechoice, stat*=*statistics*, and “↦” represents a mapping operator from critiques to item selection criteria.

**Critique**	**Item selection criteria**
Furtherstat	*Statistics* = 0↦*statistics* = 1
	*Statistics* = 1↦*ABtesting* = 1
Reducestat	*ABtesting* = 1↦*ABtesting* = 0
	*Statistics* = 1 ∧ *ABtesting* = 0↦*statistics* = 0
Excludemc	*Multiplechoice* = 1↦*multiplechoice* = 0
Includemc	*Multiplechoice* = 0↦*multiplechoice* = 1
Excludelicense	*License* = 100↦*license* = 0
Includelicense	*License* = 0↦*license* = 100

For example, if the critique *excludelicense* is specified by the user, an item supporting the selection criteria *license* = 0 will be shown.

Table 8 shows an example of a *unit critiquing session* where the initial (reference) item presented to the user is *i*_1_. The user specifies the critique *furtherstat* which requires the inclusion of additional statistic features into the survey software service configuration. Taking into account, this critique means to combine the properties of the reference item *i*_1_ (*ABtesting* = 0, *statistics* = 1, *license* = 0, *multiplechoice* = 0) with the selection criteria derived from Table 7 (i.e., *ABtesting* = 1), resulting in a new set of criteria ({*ABtesting* = 1, *statistics* = 1, *license* = 0, *multiplechoice* = 0}). There does not exist an item in {*i*_1_..*i*_5_} which completely fulfills these criteria. A related tradeoff is to choose an item as a new reference item which is mostly similar to the current selection criteria—in our case, this is item *i*_3_ which only requires the adaptation of *license* = 0 to *license* = 100. The user again specifies a critique (*includemc*) which results in a new set of criteria ({*ABtesting* = 1, *statistics* = 1, *license* = 100, *multiplechoice* = 1}), leading to the new reference item *i*_5_ which is finally accepted by the user.

**Table 8:** A simple example of a unit-critiquing session consisting of two critiquing cycles.

**Attribute**	**Ref. item (** *i*_1_ **)**	**Ref. item after cycle 1 (** *i*_3_ **)**	**Ref. item after cycle 2 (** *i*_5_ **)**
ABtesting	0	1	1
Statistics	1	1	1
License	0	100	100
Multiplechoice	0	0	1
*Unit critique*	(1) further statistics	(2) includemc	Accept

In critiquing cycle (1), the critique *further statistics* is specified on the reference item *i*_1_, in cycle (2), *includemc* is specified as a critique. After cycle (2), the user is satisfied with (accepts) the recommended item (*i*_5_).

The idea of critiquing-based recommendation is to identify an item which is similar to the current item but (in addition) takes into account the criteria specified by the critique. In our example, we have assumed that the search for a new item returns an exact match (*EIB* metric), i.e., all search criteria are fulfilled. However, the search for new reference items is often designed more flexible, for example, other attribute-level similarity metrics can be applied to determine new reference items (Lorenzi and Ricci, 2005).

#### 3.2.7 Further critiquing approaches

For demonstration purposes, we have discussed the concepts of unit critiquing in more detail. However, critiquing-based recommender systems support various alternative types of critiques (Chen and Pu, 2012; Güell et al., 2020). The idea of *compound critiques* (Smyth et al., 2004; Zhang et al., 2008) is to allow the definition of critiques which refer to more than one item property. A related example would be the compound critique *furtherstat and includelicense*, indicating the interest of a user in further statistics features also accepting (additional) license costs. A major advantage of compound critiquing is that due to the specification of multiple criteria in one critiquing cycle, significantly larger “jumps” in the itemspace are possible (Aggarwal, 2016) and—as a result—less critiquing cycles are needed to find a solution. As an extension of compound critiques, *dynamic critiques* (Reilly et al., 2004; McCarthy et al., 2005) are based on the idea of mining frequent critiquing patterns which are then presented to a user.

#### 3.2.8 Handling inconsistent search criteria

Critiquing-based recommender systems try to take into account the critiques defined by a user. Since user preferences (search criteria) typically change within the scope of a recommendation session, inconsistencies can occur (Atas et al., 2021). For example, a user is first interested in a survey software configuration without license fee resulting in the reference item *i*_1_. Then, the user wants to additionally include the *ABtesting* feature which then results in an inconsistency, since no item supports both, no license fee and *ABtesting* at the same time. From the logical point of view, such (inconsistent) search criteria are constraint sets *CRIT* = {*crit*_1_..*crit*_*q*_} and in the case of inconsistencies in *CRIT*, one option is to determine explanations (diagnoses) which indicate different options of resolving an inconsistency.^6^ Alternatively, weighting schemes are applied in such contexts meaning that “elder” critiques have a higher probability of being deleted from a critiquing history. Another alternative is the retrieval of a new reference item which is as much as possible similar to the current set of search criteria (see our example in Table 8).

#### 3.2.9 Examples of case-based recommender systems

Table 9 provides an overview of example case-based recommender applications. (Burke et al., 1996) introduce the FindMe approach to support a unit critiquing-based navigation in complex information spaces—discussed item domains are cars, videos, and apartments. The Entree system (Burke, 2000) supports unit critiquing-based recommendations in the restaurant domain, and this system is also based on the FindMe approach introduced by (Burke et al., 1996). DieToRecs (Fesenmaier et al., 2003) is a case-based recommender system that supports a similarity-based approach to case retrieval, i.e., users have to specify their requirements and select a case of relevance which is then used as a basis for further search. Recomment (Grasch et al., 2013) is a natural language-based user interface with an underlying unit-critiquing-based recommender system. (Musto et al., 2015) introduce an asset allocation strategy framework which supports the recommendation of asset classes (e.g., Euro Bond, High Yield Bond, Euro Stocks, and Emerging Market Stocks) that fulfill the goals of the investor and are basically generated on the basis of an analysis of the portfolios of similar clients (investors). Lee and Kim (2015) introduce eHeaRSS which is a case-based recommender system in the healthcare domain which helps to identify, for example, relevant doctors, depending on the current (aggregated) health record and those of similar cases. In this context, an ontology helps to assure the correctness of recommendations, for example, in terms of the proposed medical diagnosis. Feely et al. (2020) introduce a case-based recommendation approach for the recommendation of marathon training plans and race pacings based on case information about the workouts and race histories of similar runners. CEBRA (Hernandez-Nieves et al., 2021) supports the recommendation of banking products using a basic case-based reasoning recommender operating on a user's financial service profile and corresponding demographic data. Finally, the smart city initiative (Bokolo, 2021) focuses on the case-based recommendation of smart city dimensions, i.e., by taking a look at similar cities, corresponding smart city-related activities for the current city are determined in order to achieve predefined sustainability goals, for example, on the basis of the best use of technological and human resources.

**Table 9:** Overview of example case-based recommender applications.

**Recommender application**	**Recommendation domain**	**References**
RentMe	Apartments	Burke et al., 1996
Entree	Restaurants	Burke, 2000
DieToRecs	Travel plans	Fesenmaier et al., 2003
Recomment	Digital cameras	Grasch et al., 2013
Asset allocation framework	Financial services	Musto et al., 2015
eHeaRSS	Healthcare	Lee and Kim, 2015
Recommender for Recreational Runners	Training plans	Feely et al., 2020
CEBRA	Financial services	Hernandez-Nieves et al., 2021
Smart City Initiative	Smart cities	Bokolo, 2021

### 3.3 Constraint-based recommendation

The concept of constraint-based recommendation (Felfernig and Burke, 2008) is based on the idea that recommendation knowledge is represented in terms of a set of variables and a corresponding set of constraints. As already mentioned, such constraints can be defined in an extensional fashion (by just enumerating the items part of the solution space) or in an intensional fashion where the recommendation space is represented in terms of a set of logical formulae. In this context, a constraint-based recommendation task (CB-REC Task) can be defined as follows (see Definition 1).^7^

**Definition** 1. A constraint-based recommendation task (CB-REC Task) can be defined as a constraint satisfaction problem (CSP) (*V, C, R*) where *V* = {*v*_1_..*v*_*n*_} is a set of finite domain variables with associated variable domain definitions *dom*(*v*_*i*_), *C* = {*c*_1_..*c*_*m*_} is a set of constraints, and *R* = {*r*_1_..*r*_*k*_} is a set of user requirements.

Following Definition 1, an example recommendation task (*V, C, R*) based on the entries in Table 5 is as follows: *V* = {*ABtesting, statistics, multiplechoice, license*}, *dom*(*ABtesting*) = *dom*(*statistics*) = *dom*(*multiplechoice*) = {0, 1}, *dom*(*license*) = {0, 100}, *C* = {*c*_1_..*c*_5_}, and *R* = {*r*_1_: *ABtesting* = 1}, assuming that the user is interested in the *ABtesting* feature.

A constraint-based recommendation CB-REC for a given constraint-based recommendation task (CB-REC Task) can be defined as follows (see Definition 2).

**Definition 2**. A constraint-based recommendation (CB-REC) for a defined CB-REC Task is a set of tuples *REC* = ⋃_*i*_α_ ∈ *I*_{(*rank*_*iα*_, *i*_α_)} where *rank*_*iα*_ represents the recommendation rank assigned to item *i*_α_ ∈ *I* defined by a recommendation function *rf* and ∀(*rank*_*iα*_, *i*_α_) ∈ *REC*: consistent(*C*∪*R*∪*a*(*i*_α_)) where *a*(*i*_α_) denotes the variable value assignments associated with item *i*_α_.

Assuming *R* = {*r*_1_ : *ABtesting* = 1}, a CB-REC in our working example could be *REC* = {(1, *i*_5_), (2, *i*_3_)} where *a*(*i*_3_) = {*ABtesting* = 1, *statistics* = 1, *license* = 100, *multiplechoice* = 0} and *a*(*i*_5_) = {*ABtesting* = 1, *statistics* = 1, *license* = 100, *multiplechoice* = 1}.

#### 3.3.1 Ranking items

Up to now, we did not specify a function *rf* for the ranking of the items in *REC*. A simple ranking function could just count the number of supported features (see Equation 1), which would result in the mentioned recommendation ranking of *REC* = {(1, *i*_5_), (2, *i*_3_)} since the number of supported features of *i*_5_ is 3 [*support*(*i*_5_) = 3] whereas *support*(*i*_3_) = 2. In other words, item *i*_3_ is outperformed by one item resulting in a ranking of 2.^8^


(1) $$\begin{array}{l} rf\left(i_{\alpha }\right)=1+|\left\{i_{k}\in REC\left(k\neq \alpha \right):support\left(i_{k}\right)>support\left(i_{\alpha }\right)\right\}| \end{array}$$

An alternative to our simplified ranking function (Equation 1) is to introduce a utility function which evaluates utility of individual items on the basis of a pre-defined set of interest dimensions (Felfernig et al., 2006, 2018a). In our software service recommendation scenario, examples of relevant interest dimensions are *economy* and *quality* (see also Table 10).

**Table 10:** A simple utility-based evaluation scheme for recommendation candidates, for example, paying no license fee, i.e., *license* = 0, contributes to the evaluation dimension economy.

**Interest dimension**	**ABtesting**		**Statistics**		**License**		**Multiplechoice**	
	**0**	**1**	**0**	**1**	**0**	**100**	**0**	**1**
Economy	0	0	0	0	10	0	0	0
Quality	0	10	0	10	0	0	0	10

Such utility-based evaluation schemes can then be used by a utility function to determine the overall utility of individual items—see, for example, Equation (2)— where *D* represents a set of interest dimensions [in our case, *D* = {*economy, quality*}] and *eval*(*i*_α_, *d*) represents the evaluation scheme as presented in Table 10. Assuming equal importance of the two example interest dimensions [e.g., *importance*(*economy*) = 0.5 and *importance*(*quality*) = 0.5], *utility*(*i*_3_) = 0.0 + 10.0 = 10.0 and *utility*(*i*_5_) = 0.0 + 15 = 15.0, i.e., item *i*_5_ has a higher utility compared with item *i*_3_. Depending on the user-specific importance of individual interest dimensions, the resulting utility values can differ.


(2) $$\begin{array}{l} utility\left(i_{\alpha }\right)=\Sigma_{d\in D}eval\left(i_{\alpha },d\right)\times importance\left(d\right) \end{array}$$

#### 3.3.2 Dealing with inconsistent requirements

In constraint-based recommendation, it can be the case that individual user requirements do not allow the determination of a recommendation.^9^ For example, if a user is interested in the *ABtesting* and *multiplechoice* features but does not want to pay a license, i.e., *R* = {*ABtesting* = 1, *multiplechoice* = 1, *license* = 0}, no solution/item can support this set of requirements. In this example, we are able to identify two different conflicts (Junker, 2004) (see Definition 3), which are minimal sets of requirements that induce an inconsistency.

**Definition 3**. A conflict (set) *CS*⊆*R* is a set of constraints with inconsistent(*CS* ∪ *C*), i.e., no solution can be found for *CS* ∪ *C*. A conflict set *CS* is minimal if ¬∃*CS*′:*CS*′ ⊂ *CS* (subset minimality).

Conflict set minimality is important due to the fact that just one requirement needs to be deleted (i.e., relaxed) from *CS* in order to resolve the conflict. In our example, there exist two minimal conflict sets which are *CS*_1_:{*ABtesting* = 1, *license* = 0} and *CS*_2_:{*multiplechoice* = 1, *license* = 0}. To resolve the conflict *CS*_1_, a user can choose between the two options of excluding *ABtesting* (*ABtesting* = 0) or paying a *license* (*license* = 100). The same approach can be followed to resolve *CS*_2_, i.e., to either accept *multiplechoice* = 0 or accept *license* = 100.

Such conflict sets can be used in interactive recommendation settings to support users in figuring out ways from the *no solution could be found* dilemma. A set Δ of requirements is denoted as diagnosis (Reiter, 1987; Felfernig et al., 2009b, 2012) if it helps to resolve all identified conflicts (see Definition 4).

**Definition 4**. A diagnosis Δ ⊆ *R* is a set of constraints with consistent(*R* − Δ ∪ *C*), i.e., at least one solution can be found for *R* − Δ ∪ *C*. A diagnosis Δ is minimal if ¬ ∃ Δ ′:Δ′ ⊂ Δ (subset minimality).

Both concepts, i.e., *conflict sets* and corresponding *diagnoses* can be used to support users in inconsistent situations. Conflict sets are helpful in the context of *repeated conflict resolution* (for example, when purchasing a new car for the family, the preferences of individual family members change over time), and diagnoses can be used when users are interested in *quick repairs* (for example, when parametrizing an operating system, some inconsistent parameter settings need to be adapted).

#### 3.3.3 Examples of constraint-based recommender systems

Table 11 provides an overview of example constraint-based recommender applications. FSAdvisor (Felfernig and Kiener, 2005) is a financial service recommender system that proposes new financial services depending on the current financial status and requirements of the customer. In the line of FSAdvisor, VITA (Felfernig et al., 2007) is a financial service recommender system supporting sales representatives in the preparation and conduction of customer dialogs. Both systems are based on a database query-based approach implemented in AdvisorSuite, which is a development environment for constraint-based recommender applications (Felfernig et al., 2006; Jannach and Kreutler, 2007). In addition, Mortimer is a constraint-based recommender application developed on the basis of AdvisorSuite which focuses on the constraint-based recommendation of cigars. Authentic (Murphy et al., 2015) is a constraint satisfaction-based recommendation environment for supporting the achievement of energy saving goals on the basis of adapted schedules for activating household appliances. Recommendations of behavior change (e.g., postponed activation of a dishwasher to exploit more self-produced energy) are determined on the basis of energy consumption-related sensor data. Wobcke et al. (2015) introduce a P2P recommendation environment in the context of online dating scenarios where constraints (rules) are used to define baseline recommendation strategies. RecTurk (Ulz et al., 2017) is a framework for the development of constraint-based recommender applications. The underyling idea is to apply the concepts of *human computation* (Law and von Ahn, 2011) to alleviate the definition and maintenance of recommendation rules. Almalis et al. (2018) introduce a constraint-based job recommender system where constraints are used to assure defined compatibilities between job seekers and job announcements. Finally, Esheiba et al. (2021) present a constraint-based recommender system supporting the filtering-out of irrelevant product services and the ranking of the remaining relevant ones.

**Table 11:** Overview of example constraint-based recommender applications.

**Recommender application**	**Recommendation domain**	**References**
FSAdvisor	Financial services	Felfernig and Kiener, 2005
Advisor Suite	Digital cameras	Felfernig et al., 2006
Mortimer	Cigars	Zanker et al., 2006
VITA	Financial services	Felfernig et al., 2007
Authentic	Energy saving	Murphy et al., 2015
P2P framework	Online dating	Wobcke et al., 2015
RecTurk framework	Digital cameras	Ulz et al., 2017
FoDRA framework	Jobs	Almalis et al., 2018
PSS recommender	Product services	Esheiba et al., 2021

## 4 Recent advances in knowledge-based recommendation

In this section, we provide an overview of the recent advances in knowledge-based recommendation (compared with the basic approaches presented in Section 3). We discuss the following aspects: (1) Users are often in the situation of not knowing every detail about a product and—as a consequence—are not able to make a related informed decision. For example, if someone is interested in digital cameras, he/she might be able to specify preferences regarding attributes such as *resolution, maximum price, weight*, and *primary application* (e.g., landscape photography or sports) but at the same time might not be able to specify preferences regarding attributes such as *maximum frames per second* or *maximum exposure time*. In our example, users might not be aware of the usefulness of the *ABtesting* feature. Related techniques to support users in such contexts are presented in Section 4.1. (2) Given a set of user preferences, situations can occur where no solution can be identified (see also Section 3)—in Section 4.2, we introduce concepts for personalized conflict resolution. (3) In some situations, users have adaptation (reconfiguration) requirements regarding already purchased items. For example, after having purchased an online survey software license, a user detected the relevance of the *ABtesting* feature and is interested in a feature extension. Related technical solutions are presented in Section 4.3. (4) Knowledge-based recommendation is not only relevant in recommendation scenarios focusing on single users but also in scenarios where recommendations have to be determined for groups, for example, planning round-trips for tourist groups or deciding about the features to be included in a software or service. Aspects of knowledge-based group recommender systems are presented in Section 4.4. (5) In Section 4.5, we discuss further approaches focusing on the topics of hybrid recommendation, search optimization, explanations, recommendation knowledge acquisition, conversational recommendation, and explanations.

### 4.1 Recommending preference settings

There are situations where users need help in specifying preferences regarding specific item properties. Table 12 depicts a simplified example of an attribute value proposal in the context of a *case-based recommendation* scenario: the *current* user has already specified his/her preferences regarding the attributes *multiplechoice* and *statistics*. Using a nearest neighbor (NN)-based approach, i.e., determining the session with the most similar preferences, results in session *s*_3_. Consequently, we can recommend the attribute values *ABtesting* = 1 and *license* = 100 to the *current* user. The same approach to attribute value recommendation can also be applied (1) in the context of customer segmentation (recommendation) where customers have to be identified who will be interested in a new item feature (Felfernig et al., 2021) and (2) to figure out features (attributes), a customer is interested in specifying—this is relevant in scenarios where users are not interested in specifying all attributes (Mirzadeh et al., 2005; Dragone et al., 2018).

**Table 12:** Example user sessions *s*_*i*_ representing “successfully” completed recommendation sessions (e.g., a user has selected a specific item).

**Session**	**ABtesting**	**Statistics**	**Multiplechoice**	**License**
*s*_1_	1	1	0	100
*s*_2_	0	1	0	0
*s*_3_ (NN)	1	1	1	100
*Current*	?	1	1	?

In this example, user (session) *s*_3_ can be regarded as the nearest neighbor (NN) of the *current* user, i.e., both, *ABtesting* and *license* are recommended to be included.

An alternative to NN-based recommendation is to apply model-based approaches such as matrix factorization or neural networks—for related details see Erdeniz et al. (2019, 2022) and Uta et al. (2022).

Beyond case-based recommendation, such approaches to attribute value recommendation are also applied in *constraint-based recommendation* (and beyond) (Felfernig and Burke, 2008; Falkner et al., 2011; Fargier et al., 2016; Pereira et al., 2016; Temple et al., 2017). A major issue in such contexts is the fact that a recommendation of one or more attribute settings could be *inconsistent* with the underlying set of constraints (C). One way to assure consistency is to pre-determine a set of nearest neighbors and to show recommendations to users after having tested recommendation consistency (Cöster et al., 2002; Felfernig and Burke, 2008; Falkner et al., 2011; Pereira et al., 2016).

An alternative is to learn *solver search heuristics* in such a way that constraint solvers are enabled to predict user-relevant attribute value settings (Uta et al., 2022), i.e., to directly integrate recommendation knowledge into constraint solver search, for example, by applying neural networks that receive as input a set of user preferences and propose corresponding solver search heuristics [in terms of variable (value) orderings] as output. In the example shown in Table 12, variable value orderings for the not yet instantiated variables *ABtesting* and *license* would be *ABtesting* [1,0] and *license* [100,0], indicating that the solver should first try to instantiate these variables with 1 (100) before trying other instantiations. For an overview on the integration of machine learning and constraint solving, we refer to Popescu et al. (2022).

In *critiquing-based recommendation*, such attribute value recommendations could be also used in the initial preference specification phase. However, critiquing-based recommendation follows the idea of critiquing a shown reference item. Specifically, in critiquing scenarios, attribute value recommendation is “replaced” by the recommendation of critiques (McCarthy et al., 2010; Mandl and Felfernig, 2012; Murti et al., 2016; Xie et al., 2018)—see the simplified unit critique recommendation example depicted in Table 13. In this simplified example,^10^ the critiquing session most similar to the critiquing session of the *current* user (session) is *cs*_2_. This can be determined, for example, by determining the intersection of the critiquing sets of *cs*_*i*_ and *current* where |*critiques*(*cs*_1_) ∩ *critiques*(*current*)| = 1 and |*critiques*(*cs*_2_) ∩ *critiques*(*current*)| = 2. Using a nearest neighbor-based approach, critiques and item selection of *cs*_2_ can be used to recommend future critiques to the *current user* but also to predict items that will be chosen by the current user (Mandl and Felfernig, 2012).

**Table 13:** Example critiquing sessions *cs*_*i*_ representing “successfully” completed recommendation sessions.

**Session**	**Reference item**	*crit*_1_	*crit*_2_	*crit*_3_	**Selection**
*cs*_1_	*i*_1_	*i*_1_.*furtherstat*	*i*_4_.*reducestat*	-	*i*_1_
*cs*_2_ (NN)	*i*_1_	*i*_1_.*includemc*	*i*_4_.*furtherstat*	*i*_5_.*excludemc*	*i*_3_
*Current*	*i*_1_	*i*_1_.*includemc*	*i*_7_.*furtherstat*	?	?

In this example, session *cs*_2_ can be regarded as the nearest neighbor (NN) of the current user. The reference item *i*_1_ is presented in Table 4.

### 4.2 Dealing with “no solution could be found” situations

As discussed in Section 3, users of knowledge-based recommenders in some situations need support to get out of the *no solution could be found dilemma*. Approaches that can proactively support users in such contexts are conflict detection (Junker, 2004) and model-based diagnosis (Reiter, 1987; Felfernig et al., 2012; Walter et al., 2017). Such algorithms help users to understand trade-offs in the current situation and propose different options to resolve inconsistencies. Having user preference weights available, for example, in terms of explicitly defined preference weights, algorithms can be applied in different ways to determine preferred (personalized) diagnoses. For demonstration purposes, we assume the following preference ordering (see Table 14) for our example conflict sets *CS*1: {*ABtesting* = 1, *license* = 0} and *CS*2: {*multiplechoice* = 1, *license* = 0} derived from the user requirements *R* = {*ABtesting* = 1, *multiplechoice* = 1, *license* = 0}.

**Table 14:** Example preference orderings for requirements (the lower the more important the preference).

**Session**	**ABtesting**	**Statistics**	**Multiplechoice**	**License**	**Diagnosis** Δ
*s*_1_	3	2	4	1	{*ABtesting* = 1, *multiplechoice* = 1}
*s*_2_	2	3	1	4	{*license* = 0}

For example, in session *s*_1_, keeping the selected *license* type as-is has the highest priority.

Following a simple additive utility-based scheme (Felfernig et al., 2013c) using the orderings depicted in Table 14 (the lower the ordering position, the higher the importance of the related user requirement), the conflicts *CS*_1_ and *CS*_2_ would be resolved (in a personalized fashion) as follows: for the user in Session *s*_1_ (user 1), we would keep the exclusion of *license* as-is and exclude both, *ABtesting* and *multiplechoice* (1 < 3+4). Vice-versa, in the case of session *s*_2_ (user 2), we would keep the preferences regarding *ABtesting* and *multiplechoice* as-is and accept the inclusion of a license fee (1+2 < 4). In a similar fashion, such an approach can be applied in critiquing-based recommendation sessions where—on the basis of importance information about individual critiques [user sentiments about individual attributes could be available or “older” critiques could be interpreted as less important (Chen et al., 2017)]—corresponding conflict resolutions could be proposed.

### 4.3 Recommendations for reconfiguration

In scenarios where users have already purchased an item and want to extend the item afterward, recommendations for reconfigurations are required, i.e., which attributes of the original solution need to be adapted such that the new user requirements can be taken into account (Felfernig et al., 2018b; Weckesser et al., 2018). For example, if a user has already ordered a specific survey software service consisting of the feature {*statistics* = 1} (item *i*_1_) but then detects that he/she wants to include also the *ABtesting* feature, the question arises which of the initial attribute settings (user preferences) remain the same and which ones need to be adapted. In such scenarios, critiquing-based recommendation would not be the primary choice since reconfiguration is in the need of *minimal changes* to an already ordered item. In contrast, case-based reasoning can be applied by using cases (items) with an exact attribute-wise match to the already purchased item with a minimal number of needed adaptations.

If we assume the availability of a product table as shown in Table 4 and a user who has purchased item *i*_1_ and now wants to extend this configuration with *ABtesting*, this results in the search criteria {*ABtesting* = 1, *statistics* = 1, *multiplechoice* = 0, *license* = 0}. The corresponding nearest neighbor configuration is item *i*_3_ [based on the *equal is better* (EIB) similarity metric]—see Table 15. In addition to the inclusion of *ABtesting*, the selection of item *i*_3_ requires one additional adaptation which is *license* = 100. Beyond counting the number of adaptations, reconfiguration can also take into account sentiments (Chen et al., 2017): if additional features could be included without further costs or efforts, this should be preferred over reconfigurations where those features are not included.

**Table 15:** Simplified reconfiguration setting: a user has already purchased item *i*_1_ and—after some time—wants to extend this configuration by including *ABtesting*.

**Item**	**ABtesting**	**Statistics**	**Multiplechoice**	**License**
*i*_1_ (purchase)	0 (1)	1	0	0
*i*_2_	0	0	1	100
*i*_3_ (NN)	1	1	0	100
*i*_4_	0	1	1	100
*i*_5_	1	1	1	100

In constraint-based recommendation, *i* = {*a*_1_..*a*_*k*_} can be regarded as a set of variable assignments (constraints) describing the original item *i* (in our example, *i*_1_); furthermore, *R* = {*r*_1_..*r*_*l*_} is a set of reconfiguration requirements (in our example, *R* = {*ABtesting* = 1}). A diagnosis Δ⊆*i* is a set of variable assignments in *i* that need to be adapted such that a solution for the reconfiguration task can be identified (Felfernig et al., 2018b). More formally, Δ⊆*i* represents a diagnosis if consistent (*i*−Δ∪*C*∪*R*), i.e., the corresponding domain-specific constraints *C* (see Table 5) need to be taken into account (Felfernig et al., 2018b). If weights for user preferences are available (see, e.g., Table 14), reconfiguration can be personalized, i.e., for individual users having purchased the same original item, different reconfigurations could be proposed. Following the standard approach of conflict detection (Junker, 2004) and diagnosis identification (Reiter, 1987), a reconfiguration can be determined the same way as shown in the context of identifying minimal sets of inconsistent requirements. If a user has originally selected (purchased) item *i*_1_, a corresponding reconfiguration requirement *R* = {*ABtesting* = 1} would induce the (singleton) conflicts *CS*_1_: {*ABtesting* = 0} and *CS*_2_: {*license* = 0}, resulting in a corresponding reconfiguration {*ABtesting* = 1, *statistics* = 1, *multiplechoice* = 0, *license* = 100} (Felfernig et al., 2018b).

### 4.4 Knowledge-based recommendation for groups

Group recommender systems (Pessemier et al., 2017; Felfernig et al., 2024b) are typically built on the top of single user recommender systems following one of the approaches of (1) *aggregated predictions* or (2) *aggregated models*. With aggregated predictions, the items recommended to individual group members can be merged. In the context of aggregated models, the preferences of individual group members are merged, resulting in a group preference model which is then used to determine recommendations.

In *case-based recommendation* for groups, the determination of group recommendations can be based on two different approaches. With *aggregated predictions*, in a first step, relevant recommendations would be determined for each user (see Table 16). In the following, these individual recommendations (items) have to be aggregated, according to a specific aggregation strategy. For example, if we apply *majority voting*, the overall recommendation for the group would be item *i*_5_ since it has received more recommendations compared with other recommendation candidates.

**Table 16:** Example of user-individual item recommendations as a basis for determining a group recommendation using the *aggregated prediction* approach (with majority voting, *i*_5_ gets recommended).

**User**	**Item**
*u*_1_	*i*_5_
*u*_2_	*i*_4_
*u*_3_	*i*_1_
*u*_4_	*i*_5_

If the recommender system is based on *aggregated models* (see Table 17), user-individual preferences have to be aggregated into a group model which can then be used for determining recommendations. In the example of Table 17, the overall case-based group recommendation would be item *i*_3_ since this one exactly represents the preferences of the determined group model.

**Table 17:** Example of a group recommendation based on the *aggregated model* approach resulting in the recommendation of item *i*_3_.

**Session**	**ABtesting**	**Statistics**	**Multiplechoice**	**License**
*u*_1_	1	1	0	100
*u*_2_	0	1	0	0
*u*_3_	1	1	1	100
Group model	1	1	0	100

We want to emphasize that following the approach of *aggregated models* can result in situations where no exact match between the attribute settings in the group model and corresponding item properties is possible. However, in such cases, the applied similarity functions will recommend items most similar to the preferences stored in the group model.

If a *critiquing-based recommendation* approach (McCarthy et al., 2006) is used, an *aggregated prediction* approach can be implemented in such a way that each group member completes an individual critiquing process, and the resulting items are then merged by an aggregation function. Following an *aggregated models* approach, individual critiques can be merged, for example, after one or more critiquing cycles, in order to show a new reference item (to individual group members) which is regarded as current recommendation for the whole group—see also McCarthy et al. (2006) and Felfernig et al. (2024b).

In *constraint-based recommendation*, in both cases, i.e., aggregated preferences and aggregated models, a group recommender would have to aggregate the preferences (attribute settings) of the individual group members (Felfernig et al., 2024b). Again, different aggregation approaches on the attribute level are possible—if we follow the goal of achieving fairness (Le et al., 2022), for example, in terms of a nearly equal number of accepted compromises per group member, a corresponding optimization (minimization) problem needs to be solved (see Equation 3). In this context, *p*(*u*_α_) denotes the preferences of group member *u*_α_, *rec* denotes the determined group recommendation, and *diff*(*p*(*u*_α_, *rec*)) denotes the number of preference tradeoffs to be accepted by group member *u*_α_. This is a group recommendation that takes into account the aspect of *fairness* minimizing tradeoff distances among individual group members.


(3) $$MIN\leftarrow \Sigma_{\{u_{i},u_{j}\}\subseteq Users}\;|diff\;(p(u_{i},rec)-diff\;(p(u_{j},rec))|$$

### 4.5 Further aspects

#### 4.5.1 Hybrid recommendation approaches

The knowledge-based recommendation approaches discussed up to now have—as a central element—an item assortment (i.e., product catalog) or a knowledge base that describes the item assortment in an intensional fashion. Knowledge-based recommendation can also be applied in hybrid recommendation scenarios taking over the role of a supportive/complementary component (Burke, 2002). In many cases, knowledge-based recommender systems take over the role of being responsible of checking domain-specific properties, for example, when recommending medical services, it must be assured that the medical item is compatible with the current state of health of a patient. Furthermore, knowledge bases (e.g., in terms of ontologies and knowledge graphs) can be applied, for example, as a basis for similarity metrics that are able to take into account knowledge structures (Bahramian and Ali Abbaspour, 2015; Colombo-Mendoza et al., 2015; Wang et al., 2019a,b; Sun et al., 2020; Zhu et al., 2020; Sha et al., 2021).

#### 4.5.2 Search optimization

Performance optimization can become an issue specifically in the context of constraint-based recommendation and related conflict detection and diagnosis problem solving (Reiter, 1987; Junker, 2004; Felfernig et al., 2012; Le et al., 2023). Improving the efficiency of constraint solving primarily depends on the ability to select optimal heuristics depending on the given search problem. Optimizing search efficiency is often based on the application of machine learning. A detailed overview of integration scenarios for constraint solving and machine learning is given in the study by Popescu et al. (2022). Specifically in the context of constraint-based recommendation, multi-criteria optimization becomes an issue since (1) constraint-based recommenders are typically applied in interactive scenarios with the need of efficient solution search and (2) at the same time, solutions must be personalized, i.e., take into account the preferences of a user. Thus, constraint-based recommendation is an important application field with the need of an in-depth integration of knowledge-based reasoning and machine learning (Uta et al., 2022).

#### 4.5.3 Knowledge acquisition and maintenance

This is an important aspect specifically in the context of constraint-based recommendation where recommendation knowledge is encoded in terms of constraints, rules, or database queries (Jannach and Kreutler, 2007; Felfernig et al., 2009a, 2013b). It must be ensured that the defined constraints correspond with real-world constraints, i.e., reflect the item domain and recommendation knowledge. The related phenomenon of the *knowledge acquisition bottleneck*, i.e., significant communication overheads between domain experts and knowledge engineers, is still omnipresent when applying such knowledge-based systems. Related research efforts focus on the topics of *automated testing and debugging, human-centered knowledge acquisition*, and *deep models of knowledge understanding*. Automated testing and debugging focuses on the application of model-based diagnosis where pre-defined test cases are used to induce conflicts in the knowledge base which are then resolved on the basis of diagnosis algorithms—see, for example, Le et al. (2021). Understanding the complexity individual knowledge structures can also help to increase the maintainability of knowledge bases—this topic includes the aspects of cognitive complexities of knowledge structures (Felfernig et al., 2015) and related knowledge structuring, for example, in terms of the ordering of constraints in a recommendation knowledge base (Felfernig et al., 2013a). Finally, human-computation-based knowledge acquisition (Ulz et al., 2017) and user query recommendation (Daoudi et al., 2016) make knowledge definition accessible to domain experts by asking simple questions, for example, *given the user requirements of* *statistics* = 1 *and* *license* = 100*, which items can be recommended?* From the answers to such questions, a recommendation knowledge base can be generated (Ulz et al., 2017).

#### 4.5.4 Conversational recommendation

Following the characterizations of Christakopoulou et al. (2016), Wu et al. (2019),; Cordero et al. (2020), Dong et al. (2020), Zhou et al. (2020), and Jannach et al. (2021) conversational recommender systems are interactive systems supporting users in the navigation of the item space in an efficient fashion and recommend items based on a systematic approach of preference elicitation. Knowledge-based recommenders can be regarded as conversational since users are guided through a preference elicitation dialog (Zou et al., 2020). However, further types of conversational recommender systems exist which extend existing approaches specifically with more flexible types of preference elicitation using, for example, natural language dialogs (Grasch et al., 2013), chatbot technologies (Tazl et al., 2019), and specific interfaces based on large language models (Dai et al., 2023).

#### 4.5.5 Explanations in knowledge-based recommendation

In the context of recommender systems, explanations can have various goals such as persuading users to purchase an item, to increase the level of trust in a recommendation, or simply to extend a user's item domain knowledge (Tintarev and Masthoff, 2012). In this context, basic explanation types are the following: (1) *why* explanations help a user to understand the reasons why a specific item has been recommended. In the context of single-shot recommendation approaches such as collaborative filtering and content-based filtering, explanations are directly related to the used algorithmic approach. For example, *this item is recommended, since similar users also liked it* or *this item is recommended, since you liked similar items in the past*. Answering such *why* questions in the context of knowledge-based recommendation means to relate recommendations to user preferences, for example, *this camera is recommended since it includes a high frame rate per second which corresponds to your requirement to be able to perform sports photography on a professional level*. This example also shows that recommendation dialogs do not necessarily include technical item properties but could also support scenarios where users specify *high-level preferences* (Felfernig et al., 2006) which are then translated into technical properties by the recommender system. (2) *Why not* explanations help users to understand in more detail why no solution could be found for their requirements. In this context, conflicts (Junker, 2004) help to understand, for example, individual incompatibilities between user requirements, whereas diagnoses (Reiter, 1987; Felfernig et al., 2012) are a general proposal (explanation) for resolving an inconsistency. (3) *How* explanations are more related to specific aspects of a recommendation process, for example, when using a utility-based approach for item ranking (Felfernig et al., 2006), explanations can take into account corresponding weights to explain how a recommendation has been determined, for example, *since you have ranked the priority of the feature fps (frame rate per second) very high, item X is the one which is ranked highest since it received the highest utility value on the basis of our evaluation function*. *How* explanations play a major role in the context of group recommendations—specifically to achieve goals such as fairness and consensus (Tran et al., 2023; Felfernig et al., 2024b). An example of taking into account such aspects is the following: *since the preferences of user X have not been taken into account for already 3 months, the current restaurant recommendation specifically focuses on taking into account the preferences of X*.

## 5 Research directions

Basic insights from our analysis are the following. A major strength of knowledge-based (specifically constraint-based) recommenders is their ability to enforce domain-specific constraints. These systems are useful when recommending and explaining high-involvement items such as cars and financial services. A major disadvantage is “setup” efforts that are needed to predefine the recommendation knowledge in terms of product properties, product catalogs, similarity metrics, and constraints. There are many new developments in *knowledge-based (recommender) systems* focusing on a “deep” integration with *machine learning*, thus helping to unify the two worlds (Popescu et al., 2022). However, there are still a couple of research directions that are of interest—see the following discussion.

### 5.1 Integrating knowledge-based systems with machine learning

Specifically in constraint-based recommender systems, a major line of research focuses on the integration of concepts and techniques from machine learning into constraint-based reasoning, for example, by learning variable and variable value ordering heuristics (Popescu et al., 2022; Uta et al., 2022). This way, recommendation functionalities can be directly integrated with constraint solving. An issue for future research is to analyze alternative integrations, for example, in which way constraint-based reasoning can be integrated into machine learning—such a goal can be achieved using neuro-symbolic AI concepts (Monroe, 2022).

### 5.2 Diagnosis performance optimization

A major runtime performance bottleneck in knowledge-based recommenders are conflict detection and diagnosis algorithms which operate on the basis of individual consistency checks (Jannach, 2006). A related topic for future research is to intensively integrate machine learning techniques that can help to reduce the number of irrelevant consistency checks as much as possible and thus help to improve runtime performance of conflict detection and diagnosis.

### 5.3 Cognitive aspects of preference elicitation

Human decision making is amenable to different types of biases with a potential negative impact on decision quality (Atas et al., 2021). Recommender user interfaces and corresponding preference elicitation dialogs have to take this aspect into account. A major issue for future research is to understand in detail in which way recommender user interfaces can have an impact on decision processes and how to counteract decision biases in such contexts.

### 5.4 Evaluation metrics for knowledge-based recommenders

Current approaches to evaluate recommender systems primarily focus on the aspect of prediction quality and user acceptance (Uta et al., 2021). However, recommender systems also have a huge impact on how customers perceive items, which items are selected, and—as a consequence—which items have to be produced. This way, recommender systems have to be evaluated with regard to various additional aspects such as the visibility of items in recommendation lists (e.g., how often is an item highly-ranked) and restrictiveness of specific features in recommendation dialogs (e.g., are there too few items that support specific feature combinations). Initial related work on these aspects can be found in the study by Lubos et al. (2023).

### 5.5 Consequence-based explanations

Explanations in recommender systems focus on reasons as to why specific items have been recommended, why no solution could be identified, and how items have been determined (Friedrich and Zanker, 2011; Tintarev and Masthoff, 2012). Future research should focus more on the generation of explanations that take into account the consequences of choosing specific items, for example, in the context of investment decisions, a consequence-based explanation could be of type *since you prefer to purchase a quite expensive car, this will have an impact on both, the quality of living (in terms of having a smaller house) and in terms of being able to have money for holidays*.

### 5.6 Integration of large language models

The application of large language models (LLMs) in knowledge-based recommender systems (and beyond) starts to play a major role in the context of various tasks such as preference elicitation (e.g., in terms of building semantically-enriched user models) and explanations, for example, by identifying the best explanation strategy in specific recommendation contexts (see Table 18—the examples have been generated with ChatGPT 3.5).^11^

**Table 18:** Examples of LLMs in knowledge-based digital camera recommendation.

**Scenario**	**Example (prompt)**	**LLM response (excerpt)**
Flexibly explaining physical properties	Why does a lower-resolution camera often provide a higher image quality?	In some cases, lower-resolution cameras have larger individual pixels. Larger pixels can capture more light, which can contribute to better image quality, especially in low-light conditions.
Flexible item comparisons	What is the most important difference between the Canon EOS 5d mark IV and the 5d mark II in terms of image quality?	The Mark IV has a wider ISO range and generally better low-light performance compared to the Mark II. The increased resolution does not sacrifice sensitivity, and the Mark IV is capable of producing cleaner images at higher ISO settings.

The examples already show the high flexibility in providing explanations to recommender users.

### 5.7 Sustainability aspects of recommender systems

Recommender systems can play a major role in achieving individual sustainability goals. These systems can help, for example, to systematically optimize energy consumption strategies in households, optimize resource reuse in supply chains, and improve personal wellbeing (Murphy et al., 2015; Felfernig et al., 2023b,c). A major issue for future research is to more intensively integrate psychological approaches such as nudging (Thaler and Sunstein, 2021) into recommendation dialogs and to develop new algorithms which help to increase the selection share of sustainable items.

### 5.8 Generalizability to other knowledge-based systems

The concepts discussed in this article can be applied to other systems beyond knowledge-based recommender systems. For example, the concepts of personalized constraint-based recommendation and corresponding diagnosis concepts are also applicable in the context of configuration and re-configuration for highly-variant and complex products and services and also in the context of scheduling and re-scheduling (Felfernig et al., 2014).

## 6 Conclusion

With this article, we provide an overview of major concepts of the knowledge-based recommender systems extending existing overviews with new technological developments. In this context, we provide working examples that help to understand the underlying techniques and algorithms in more detail. Finally, as a result of our literature analysis, we discuss different research directions in knowledge-based recommendation which will help to stimulate further related research.

## Author contributions

MU: Conceptualization, Data curation, Formal analysis, Investigation, Project administration, Software, Validation, Writing – original draft, Writing – review & editing. AF: Conceptualization, Methodology, Supervision, Validation, Writing – original draft, Writing – review & editing. V-ML: Conceptualization, Validation, Writing – review & editing. TT: Validation, Writing – review & editing. DG: Validation, Writing – review & editing. SL: Validation, Writing – review & editing. TB: Validation, Writing – review & editing.

## Footnotes

^1^ https://scholar.google.com/

^2^ https://www.researchgate.net/

^3^ https://www.sciencedirect.com/

^4^ https://link.springer.com/

^5^ https://www.elsevier.com/

^6^For details, see the following discussion of constraint-based recommender systems.

^7^For simplicity, we omit a differentiation between variables describing user preferences and those describing item properties and different constraints between these variables—for related details, we refer to Felfernig et al. (2006) and Felfernig and Burke (2008).

^8^In this context, *license* is not regarded as a feature.

^9^In critiquing-based recommendation, such situations occur if search criteria become inconsistent.

^10^For simplicity, all critiquing sessions have the same number of critiques, however, in real-world scenarios, the number of critiques can very significantly between different critiquing sessions.

^11^ openai.com
